# Households Health Expenditure in Interannual Correlation With Public Health Expenditure in Greece

**DOI:** 10.3389/fpubh.2020.00448

**Published:** 2020-10-02

**Authors:** Stavroula Zikidou, Stamatina Hadjidema

**Affiliations:** Department of Economics, University of Piraeus, Piraeus, Greece

**Keywords:** healthcare spending, Greece, recession, economic adjustment, impact, household budget survey (EOP), multiple cointegration, household health expenditure, C32, D15, I12

## Abstract

The aim of this article is to investigate the relationship between the public and the private health expenditure (macroeconomic and microeconomic approach) over time and within the recession and austerity period in Greece, in order to find out whether the strict Memorandum health policies pass, influence, or go along with the health expenditure to the final consumer, i.e., the health services user. In this context, by using econometric tools, such as multiple regression and cointegration analysis on the raw microdata of Household Budget Surveys from 1987/88 up to 2018, as well as by using data of public expenditure of Organization for Economic Co-operation and Development–Health Statistics 2019 in the Stata version 13, the study compares the variation of household and public health expenditures before and after the financial crisis in Greece and also examines the correlation between the two variables. The analysis demonstrated that the Greek household health expenditure (HHE) was rapidly increasing during the period 1988–2008, and afterward, it started decreasing. Moreover, the total private and the total public health expenditures seem to have a bidirectional long-run relationship and significant cointegration. The same was observed regarding the public expenditure and household medical services expenditure, as well as pharmaceuticals. Furthermore, the results indicate that over the years of recession, the monthly HHE decreases, due to the confiscation of middle-class income, which led to consumerism restrictions. However, as households are now spending a bigger portion of their shrunken income for health (as health is an inelastic commodity), HHE, as a proportion of total private expenditure, has eventually risen.

## Introduction

The study of health expenditure over time is a difficult task, because it has to be considered the fact that health is a product of inelastic demand and also that the public sector has a role of “payer,” and therefore, it has the power to regulate the market prices. Financial fluctuations can affect the providers, the users, and, ultimately, the population's health.

The health sector in Greece, after a period of growth within the first decade of the millennium, when total expenditure on health [% gross domestic product (GDP), (1)] exceeded 10% (above the European Union average), the following years it began to moderate. After signing the Memorandum of Understanding in 2010 (2), a number of urgent expenditure restricting measures and structural reforms were imposed to the Greek health sector and, in particular, to pharmaceutical sector. The last one seems to significantly contribute (negatively) to both deficit and public debt, due to the excessive public spending and the lack of control in both the volume and the cost of prescriptions.

Therefore, in May 2010, pharmaceutical industry became the focus of fiscal consolidation, and it was one of the main areas of intervention, in order to reduce public pharmaceutical expenditure to 1% of GDP, thus approaching the European average (3). As a result, public pharmaceutical expenditure decreased by 60.8% between 2009 and 2014, reaching €2billion in 2014 against €5.1 billion in 2009 (4).

Coupled with the economy contraction, the health expenditure in Greece has also been reduced proportionally from 9.8% of GDP in 2008 to 8.1% in 2014 (5). Reduced health costs, which were required under the austerity program, have been criticized, because they do not contain specific provisions to safeguard the National Health Service (NHS) (5). The NHS was established in the 1980s, as part of the national compulsory social security program, through which most of the Greek inhabitants have been cared. However, as mentioned above, Greece has failed to control health spending between 2000 and 2009, and the health budget deficit of the country reached €50 billion (6). Consequently, at the beginning of the crisis, the health sector was set to be a priority by the Troika, having contributed significantly to the economic derailing of the country.

On top of that, there is a “hidden” financial burden of at least 27%, according to Souliotis et al. (7), which influences negatively the living conditions of the households, which is not reported as purchasing ability or cost of living: the informal (under-the-table) payments, as a common reimbursement method for the health care services in order to gain access, to reduce waiting time, to receive a higher quality of services or out of gratitude, and so on (7). This survey also reveals that, because of severe financial pressure, there is a growing unwillingness of citizens to pay informally and that there is an increasing demand for these payments as a prerequisite for access to services or to redeem services provided. Apart from this, a more recent survey concludes that more than 60% of the health care incidents involved informal payments in Greece (8). Despite near-universal coverage of the population by public health insurance, informal payments are widespread and a major source of inequity and inefficiency in the Greek health care system (9).

The present study examined the level of the public health expenditure and the private Greek household health expenditure (HHE) within the period 1988–2018. Additionally, the existence of interaction between the public and the private expenditure was examined by using the method of cointegration, in order to determine whether a change in the Greek public health spending has ultimately the same behavior with the variation of the private HHE. The survey results will provide an enlightening insight into the evolution of health expenditure in Greece for the past 30 years, and the impact of the financial crisis will be determined, through the evolution of the costs.

## Materials and Methods

In this study, preform (meta) data were derived from the Greek Statistical Authority (HEL.STAT.), concerning the private health expenditure from the Household Budget Survey (HBS) for the years 1987, 1994, 1999, and 2004, as well as annually data from 2008 up to 2018. It should be borne in mind that HBSs are carried out randomly and at a regular basis^1^, by HELSTAT, throughout Greek territory. Through these surveys, information on the consumer spending, income, housing facilities, consumer durables and household socioeconomic characteristics, and household members was collected. In regard to the health expenditure, data collected referred to the HBS records, inter alia, households' responses to pharmaceuticals expenditure-treatment-equipment devices and non-hospital medical and hospital care services, not individually, but on household level, because the household in-charge person reflects the costs and consumer behaviors of the entire household. Additionally, data were drawn from the Organization for Economic Co-operation and Development (10) on the level of public expenditure over time as a percentage of GDP. Health spending refers to the final consumption of goods and the use of health services (i.e., current health expenditure), including the personal therapeutic care (curative care, rehabilitation, long-term nursing care, ancillary health care services and medical products, prevention, and public health services, as well as health care administrative costs, excluding investment and research–educational costs). It should be noted that the HBS results are produced in accordance with the relevant International Classification Systems, as seen below, for the category of Health.

**Table:** Description of Target Variables for Microdata Files.

Var N°	Variable name	Format
HE06	Health	INT 14
HE06.1	Medical products, appliances, and equipment	INT 14
*HE06.1.1*	*Pharmaceutical products (ND)*	*INT 14*
HE06.1.1.1	Pharmaceutical products	INT 14
*HE06.1.2*	*Other medical products (ND)*	*INT 14*
HE06.1.2.1	Other medical products	INT 14
*HE06.1.3*	*Therapeutic appliances and equipment (D)*	*INT 14*
HE06.1.3.1	Therapeutic appliances and equipment	INT 14
HE06.2	Outpatient services	INT 14
*HE06.2.1*	*Medical services (S)*	*INT 14*
HE06.2.1.1	Medical services	INT 14
*HE06.2.2*	*Dental services (S)*	*INT 14*
HE06.2.2.1	Dental services	INT 14
*HE06.2.3*	*Paramedical services (S)*	*INT 14*
HE06.2.3.1	Services of medical analysis laboratories and X-ray centers	INT 14
HE06.2.3.2	Services of medical auxiliaries	INT 14
HE06.2.3.3	Other nonhospital services	INT 14
HE06.3	Hospital services	INT 14

Also, HBSs for the years 1987/1988, 1993/1994, 1998/1999, and 2004/2005 covered all the areas of the country (urban, semiurban, and rural) with a sample size from 6,000 to 6,800 households. The duration of the aforementioned surveys was 1 year. From the year 2008, taking into consideration the national needs for the Consumer Price Index compilation, because the main purpose of the HBS is to determine in detail the HE pattern in order to revise the Consumer Price Index and in order to have higher reliability for being able to produce comparable statistics used by the National Accounts Division, the annual and continual conduct of the survey was decided with a sample of ~4,000 households in the whole Greek territory.

As regards the accuracy of the data, sampling and non-sampling errors occurred, due to the fact that the HBS is a sampling survey. The sampling errors are depicted by estimating the coefficient of variation for the main survey variables and their values proved to be within the acceptance limits. The non-sampling errors were divided into non-response errors, elaboration errors, and measurement errors. During the previous years, the overall accuracy of the survey was good enough.

Data analysis was performed via Stata software, version 13, and for the research purposes, the technique of cointegration analysis was used.

The vast majority of chronological series of economic variables are not characterized by stagnation. Applying a simple regression, just to identify the possible correlation between two or more variables, can lead to the phenomenon of malicious or apparent or spurious regression, as proposed by Granger and Newbold (11). It is very difficult to analyze time-series data. Spuriosity is an issue. A correlation may arise between them (apparent), which, as the authors argued, may be due to the existence of short-term trends, which is treated by applying first differences in time-series to turn them into time-series characterized by stagnation. The concept of integration came to eliminate this phenomenon (12). In non-stationary time-series that show the same trend or otherwise “move together,” the results of regression may not be fictitious, so in this case the usual conclusions based on statistics *t* and *F* may apply. When there is a causal relationship, such as expected, for example, between variables of income and health expenditure, the two variables will not be deviated for a long time, even though they both increase; i.e., they have a tendency and are therefore non-stationary. This “synchronization” of non-stationary time-series is the basic idea behind the concept of integration, where two or more variables move in the same direction in the long run, i.e., there is a long-term equilibrium relationship among variables, without necessarily to apply also in the short term (13).

According to the aforementioned author, two or more non-stationary variables are linked to a long-term relationship, in the sense that they “move together in time” showing the same trend. For example, if the probabilistic linear relationship of two non-stationary variables with a degree of completion of 1 is characterized by a degree of completion of 0 (stationary), then the two time-series are cointegrated, in a sense that the variable describing their relationship moves long around a point of equilibrium.

All prices in the tables/graphs hereof are in euros (€), unless otherwise defined. It should be mentioned that Greece adopted the euros in 2001, after irrevocably fixing the conversion rate on June 2000 at €1 = 340.75 drachmas. Then, on January 1, 2002, euro banknotes and coins were launched and put into circulation within the transition period at the end of February 2002 and onward. All data of HBS before this currency changeover, that is, HBS 1988, HBS 1994, and HBS 1999, for the purpose hereof, were officially given by HEL.STAT. converted to euros.

Furthermore, HBS depicts the average monthly expenditure of the whole household, so prices in the tables/graphs hereof refer to average monthly health expenditure of the whole household. There was no year chosen as a reference year for inflation observations, because the research was based in current prices. It should be noticed that the inflation rates (average consumer prices) during the period 1988–2018 ranged between 20.3 (1990) and −1.4 (2014), and more specifically during the crisis period 2008-2018 the annual percent change ranged between 4.7 (2010) and −1.4 (2014) (14).

### Cointegration Hypothesis

A set of time-series cointegrates, when there is a linear combination of these time series, which are stationary, cannot present a stochastic trend. The linear time-series combination is the equation of cointegration that represents the long-term equilibrium relationship among these sequences. For the purposes of a cointegration test, variables must be stationary at the same level. A cointegration test was performed via the Johansen test (15), in order to examine whether there is a cointegration between the variables. The Johansen test was chosen because many authors agree that it is an improvement over the Engle–Granger test and Stock & Watson's test (in Introduction to Econometrics). Furthermore, it avoids the issue of choosing a dependent variable, as well as issues created when errors are carried from one step to the next. As such, the test can detect multiple cointegrating vectors, and it is more appropriate than Engle–Granger for multivariate analysis. Another desirable property is that the Johansen test treats every test variable as endogenous variable (16).

We therefore define the following hypothesis:

- Ho: There is no cointegration between variables.
- Ha: There is cointegration.

If time series are co-integrated, it means that, in the long-term, variables move together on the same direction without however, meaning that in the short-term the same phenomenon will occur. The test shows if there are, and how many, linear cointegration relations between the controlled variables. The link between the short- and long-term relationships of variables is performed by the existence of cointegration, as a prerequisite for assessing the Vector Error Correction (VEC) model. If the findings show that in our model there is, indeed, a cointegration relationship, it allows the assessment of the VEC model. Hence, VEC model is used, because we want to simultaneously examine the results of the regressions relating to the linear equations.

All assays were performed in 5% level of significance (α = 0.05).

## Results

### Descriptive Presentation of the HHE Categories

Table 1 portray the longitudinal data on the average monthly household expenditure for medicines, therapeutic appliances, and equipment. The results are given both for the total average cost and their subcategories. All prices are in euros (€). Furthermore, it should be noted that until 2008 no data were available regarding these three cost categories separately.

**Table 1:** Average monthly expenditure on medicines, therapeutic apparatus, and equipment of the Greek households, during the period 1988–2018, in euros.

**Year**	**Medicines, therapeutic apparatus, and equipment**	**Pharmaceutical products (1)**	**Other medicines (2)**	**Therapeutic apparatus and equipment (3)**
1988	3.56	—	—	—
1994	10.4	—	—	—
1999	17.6	—	—	—
2004	25.54	—	—	—
2008	33.02	27.36	1.50	4.16
2009	31.25	25.83	1.97	3.44
2010	32.23	27.44	1.87	2.91
2011	30.84	25.33	1.73	3.77
2012	32.52	28.69	1.42	2.40
2013	37.13	33.8	1.57	1.76
2014	39.85	35.75	2.45	1.64
2015	39.60	35.98	*0.38*	1.54
2016	39.10	35.67	*0.31*	1.18
2017	38.78	35.37	0.28	1.13
2018	38.96	35.45	0.27	1.12

*Source: HEL.STAT. (HBS metadata, 1988–2018)*.

**Figure 1:**
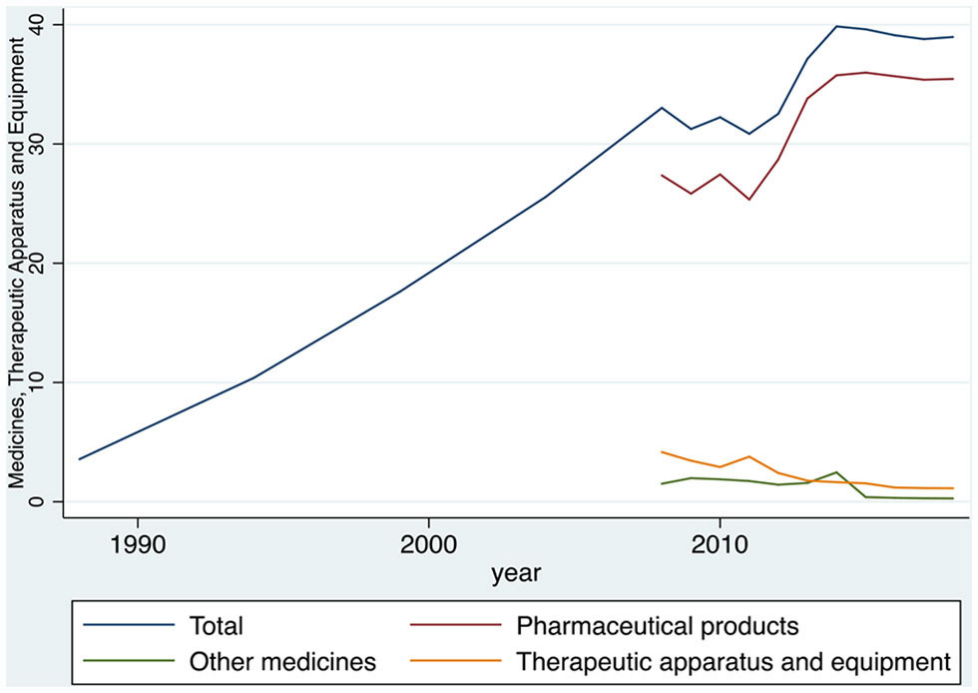
Evolution of the expenditure on medicines, therapeutic apparatus, and equipment of the Greek households, during the period 1988–2018, in euros. Source: HEL.STAT. (HBS metadata, 1988–2018).

Figure 1 shows that the average total household expenditure in medicines, therapeutic apparatus, and equipment increases rapidly from the year 1988 to the year 2018. The increase reaches 994.38%, from €3.56 in 1988 to €38.96 in 2018. Furthermore, the graph shows that within 2008–2011 (financial crisis), the average monthly household spending had a downward trend compared to previous years, whereas in the period from 2012 to 2018, it began to rise again at considerable pace. Additionally, the results showed that the biggest proportion of the category “medicines, therapeutic apparatus, and equipment” expenditure comes from the medicines expenditure. In contrast, the “other therapeutic apparatus and equipment” household expenditures seem to be stagnant and declining from 2004 to 2018.

**Figure 2:**
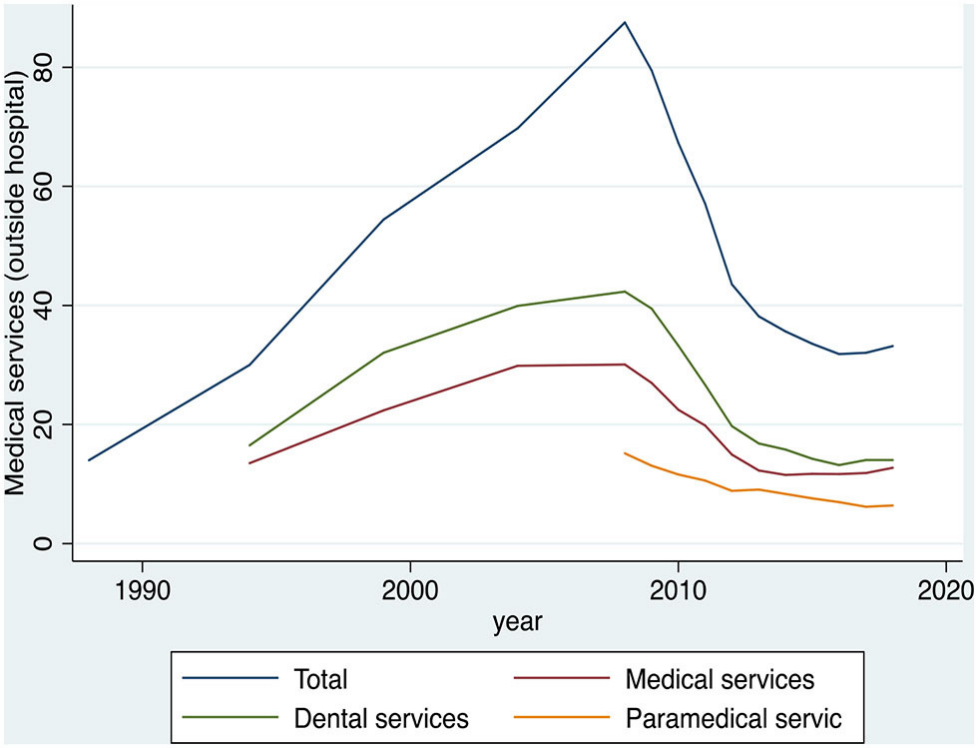
Evolution of expenditure on the medical services (outpatient) of the Greek households, during the period 1988–2018, in euros. Source: HEL.STAT. (HBS metadata, 1988-2018).

In Table 2 and Figure 2, the temporal data on the average monthly household expenditures on medical services (outpatient) are presented. The results show both the total average cost of medical services and its subcategories. Furthermore, it should be noted that there are overall figures with respect to the classification of expenditure in subcategories only after the year 2004.

**Table 2:** Average monthly expenditure on medical services (outpatient) of the Greek households, during the period 1988–2018, in euros.

**Year**	**Medical services (outpatient)**	**Medical services**	**Dental services**	**Paramedical services**
1988	14	—	—	—
1994	30	13.5	16.5	—
1999	54.42	22.38	32.04	—
2004	69.76	29.85	39.91	—
2008	87.54	30.07	42.30	15.17
2009	79.48	26.96	39.45	13.07
2010	67.30	22.45	33.25	11.59
2011	57.09	19.84	26.65	10.59
2012	43.54	14.94	19.73	8.87
2013	38.15	12.27	16.81	9.08
2014	35.63	11.52	15.8	8.31
2015	33.54	11.71	14.23	7.60
2016	31.83	11.67	13.19	6.97
2017	32.03	11.83	14.02	6.19
2018	33.18	12.74	14.02	6.41

*Source: HEL.STAT. (HBS metadata, 1988–2018)*.

Figure 2 shows that the average monthly total household expenditure on medical services was increasing from 1988 to 2008 (growth rate of 525.29%, from €14 in 1988 to €87.54 in 2008). In contrast, within the period 2008–2018, the average monthly total household expenditure on medical services appeared to have a declining trend from €87.54 in 2008, to €33.18 in 2018, which is a 62.1% decrease. Additionally, the results showed that, since the beginning of the financial crisis in 2008, the average household expenditure for medical services had a downward trend compared to the previous years. Moreover, the biggest part of expenditure for medical services derives from the dental services. In contrast, the paramedical services expenditure seems to have been stagnant and declining from 2004 to 2018, while it happens to be the smallest percentage among three subcategories of the medical expenses.

**Figure 3:**
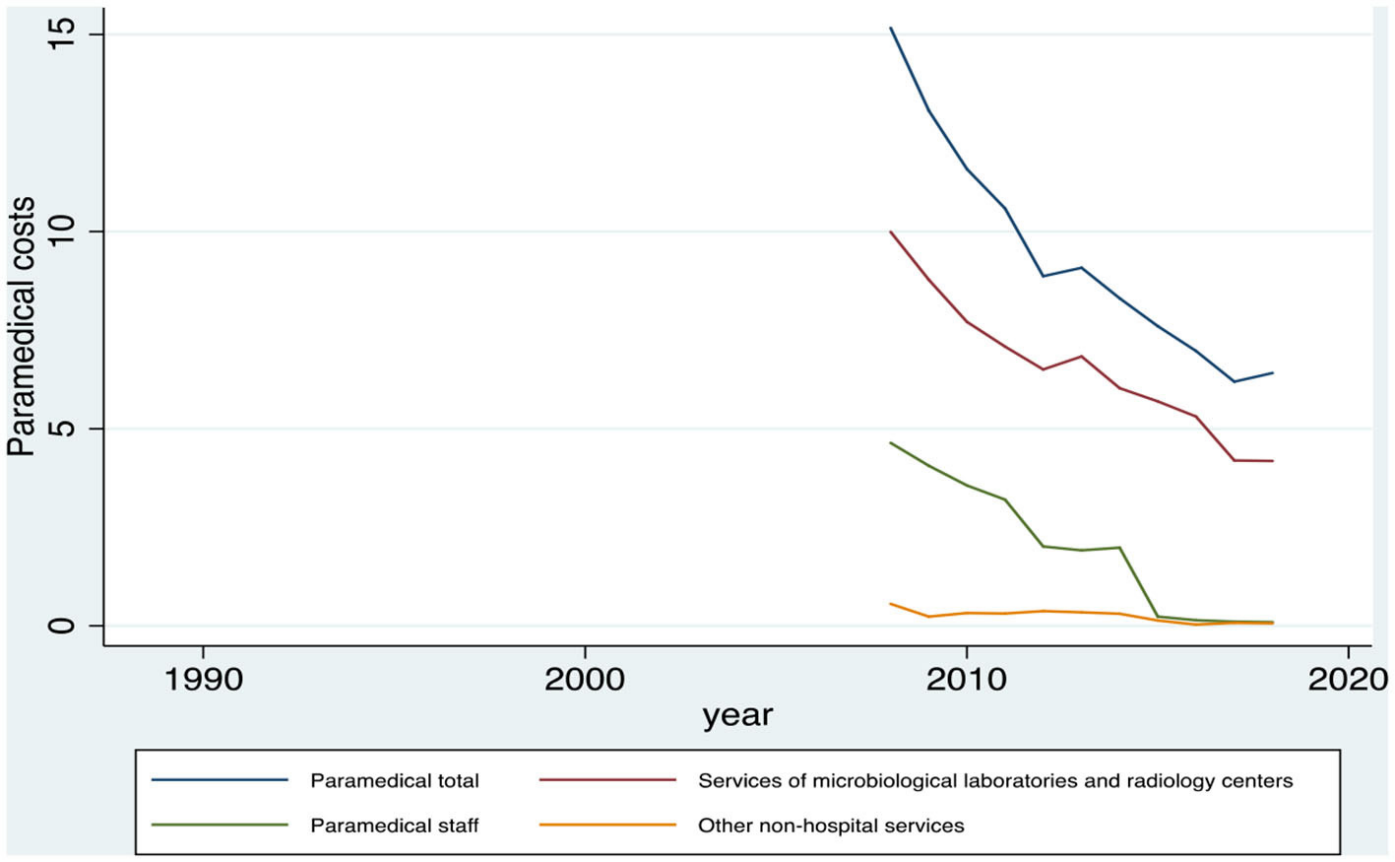
Evolution of the paramedical expenditure and the subcategories of the Greek households, during the period 1988–2018, in euros. Source: HEL.STAT. (HBS metadata, 1988-2018).

As regards the paramedical Greek household services, particularly, from HBS data for the years 1987, 1994, 1999, and 2004 and the period from 2008 to 2018, it is observed that the biggest portion of the expenditure for paramedical services is related to the microbiological laboratory services and the radiology centers, followed by the paramedical staff services, while a very small percentage is attributable to the other non-hospital services. Table 3 and Figure 3 indicate a common and constant reduction of costs in all three subcategories over the years.

**Table 3:** Average monthly expenditure on paramedical services of the Greek households, during the period 1988–2018, in euros.

**Year**	**Paramedical services**	**Microbiological laboratory services and radiology centers**	**Paramedical staff services**	**Other non-hospital services**
2008	15.17	9.99	4.64	0.55
2009	13.07	8.78	4.06	0.23
2010	11.59	7.71	3.56	0.32
2011	10.59	7.08	3.20	0.31
2012	8.87	6.50	2.01	0.37
2013	9.08	6.83	1.91	0.34
2014	8.31	6.03	1.98	0.3
2015	7.60	5.69	0.23	0.13
2016	6.97	5.31	0.14	0.03
2017	6.19	4.19	0.1	0.07
2018	6.41	4.18	0.09	0.06

*Source: HEL.STAT. (HBS metadata, 1988–2018)*.

**Figure 4:**
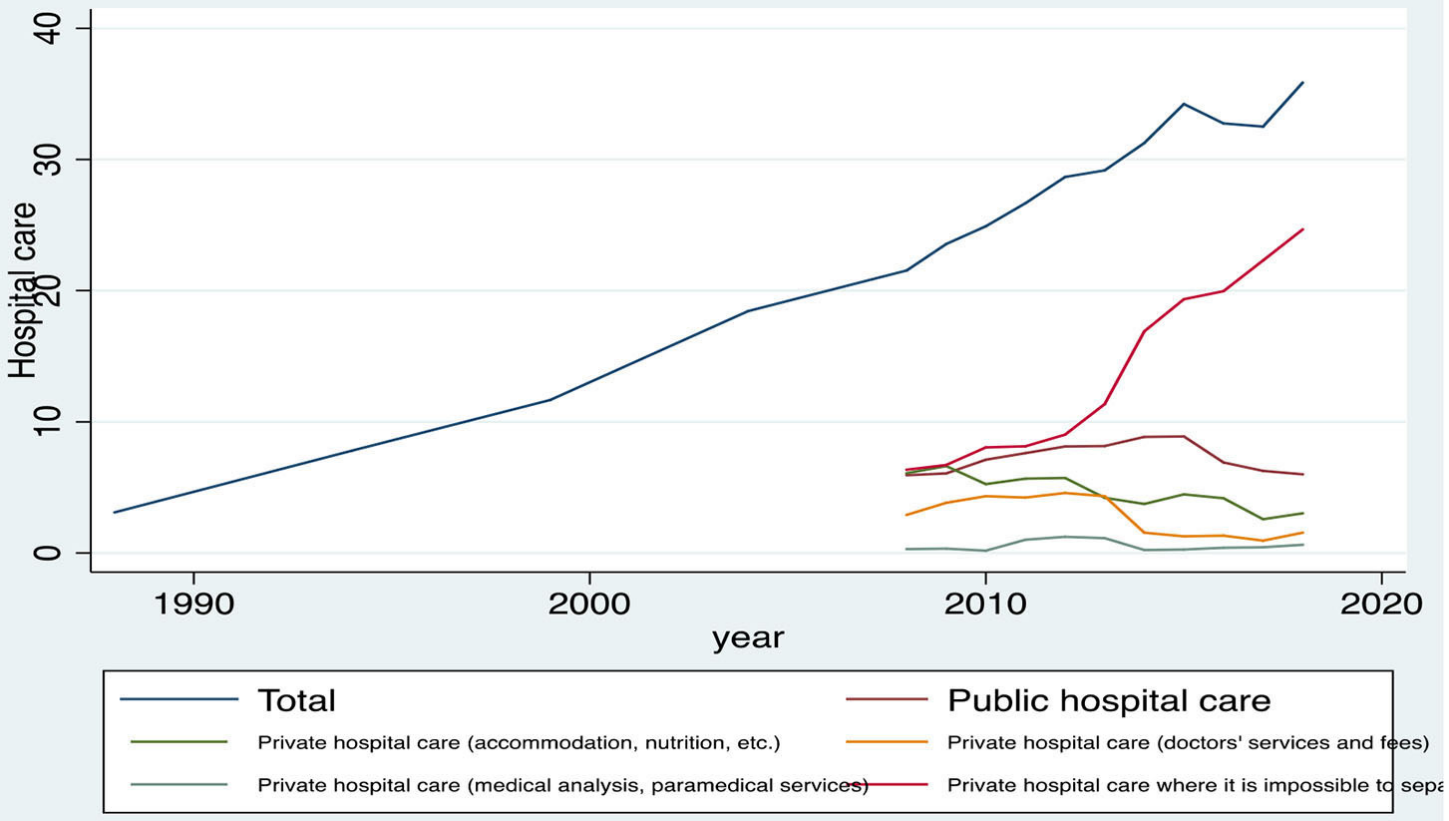
Evolution of the hospital care expenditures of the Greek households, during the period 1988–2018, in euros. Source: HEL.STAT. (HBS metadata, 1988–2018).

The next category of health expenditure refers to the hospital care, and the analytical results are presented in Table 4 and Figure 4 below. The results show that the average monthly expenditure of the Greek households for the hospital care increased steadily over the period 1988–2018. Specifically, the average cost of hospitalization increased from €3.1 in 1988 to €35.85 in 2018, which is equal to an increase of 1.056.45% (~10-fold average expenditure). As regards the subcategories of the hospital care, it is noticed that the highest average contribution to the total hospital care was the private hospital care, where it is impossible to separate accommodation services and health services (medical or paramedical) followed by the public hospital and the private hospital care (accommodation, nutrition, etc.). Regarding the subcategories of the hospital care expenditure, the most important finding is that the private hospital care expenditure, where it is impossible to separate the accommodation services and the health services (medical or paramedical), increases considerably over the years, whereas the other categories show either stagnation or decline.

**Table 4:** Average monthly expenditure on the hospital care of the Greek households, during the period 1988–2018, in euros.

**Year**	**Hospital care**	**Public hospital care**	**V1**	**V2**	**V3**	**V4**
1988	3.1	—	—	—	—	—
1994	7.8	—	—	—	—	—
1999	11.66	—	—	—	—	—
2004	18.44	—	—	—	—	—
2008	21.54	5.93	6.08	2.91	0.29	6.34
2009	23.55	6.06	6.62	3.82	0.33	6.71
2010	24.90	7.11	5.24	4.32	0.17	8.05
2011	26.66	7.62	5.67	4.22	1.01	8.14
2012	28.66	8.12	5.71	4.57	1.24	9.02
2013	29.16	8.15	4.21	4.33	1.13	11.35
2014	31.25	8.85	3.73	1.55	0.22	16.9
2015	34.22	8.88	4.47	1.27	0.26	19.34
2016	32.75	6.90	4.18	1.32	0.40	19.95
2017	32.51	6.25	2.58	0.94	0.43	22.3
2018	35.85	6	3.03	1.54	0.62	24.66

*V1 = private hospital care (accommodation, nutrition etc.), V2 = private hospital care (doctor's services and fees), V3 = private hospital care (medical analysis, paramedical services), V4 = private hospital where it is impossible to separate accommodation services and health care services (medical or paramedical)*.

*Source: HEL.STAT. (HBS metadata, 1988–2018)*.

It is also noticed that within the period 2008–2018, the average private hospital care, where it is impossible to separate the accommodation services and the health services (medical or paramedical) expenditure, increased from €6.34 to €24.66 (growth rate 288.96%), whereas the average monthly expenditure for the private hospital care (accommodation, food, etc.) decreased from €6.08 to €3.03 (reduction rate 50.16%), and the average monthly expenditure for the private hospital care (doctors' services and fees) decreased from €2.91 to €1.54 (reduction rate 47.08%).

### Descriptive Presentation of the Total Health Expenditure

**Figure 5:**
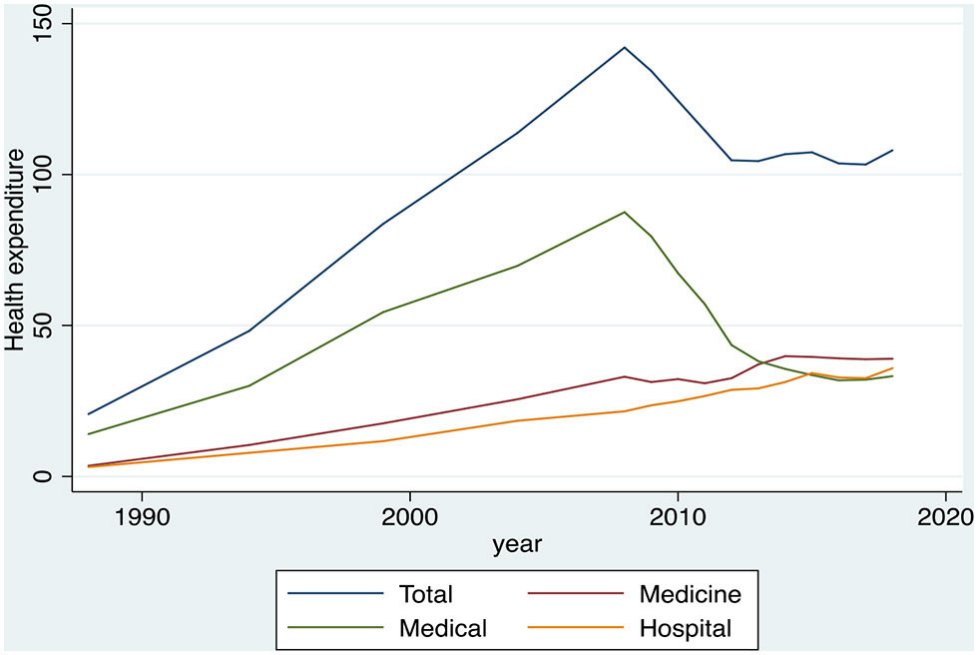
Evolution of the total health expenditures and its main subcategories (medical, pharmaceutical, hospital) of the Greek households, during the period 1988–2018, in euros. Source: HEL.STAT. (HBS metadata, 1988-2018).

Findings concerning the total HHE for the period 1988–2018 and its three main subcategories of expenditure (medical, pharmaceutical, hospital) are shown below. Figure 5 shows that the total monthly HHE in Greece had an upward trend during the period 1988–2008. Specifically, the total monthly expenditures from €20.66 in 1988 reached €142.1 in 2008, which refers to an increase of 587.8% (5-fold increase on expenditure). On the contrary, from the beginning of the financial crisis up to 2018, the average monthly total household expenditure declined from €142.1 in 2008 to €107.99 in 2018, which is a reduction of 24%. Furthermore, as follows, up to the beginning of the financial downturn in 2008, the bulk of the total health expenditure derives from the medical expenditure, whereas from 2008 onward, the medical costs had a decrease of 62.1%. Then, from 2012 onward, this kind of costs equilibrated to the pharmaceutical costs and the expenditures for hospital care. Finally, another important finding is that, after the year 2008, the spending on medical services seems to be largely affected, whereas the pharmaceutical costs and the expenditures for the hospital care appeared to have a small, but steady increase (17.99 and 66.43%, respectively).

### Descriptive Presentation of the Public Health Expenditure as a Percentage of GDP

**Figure 6:**
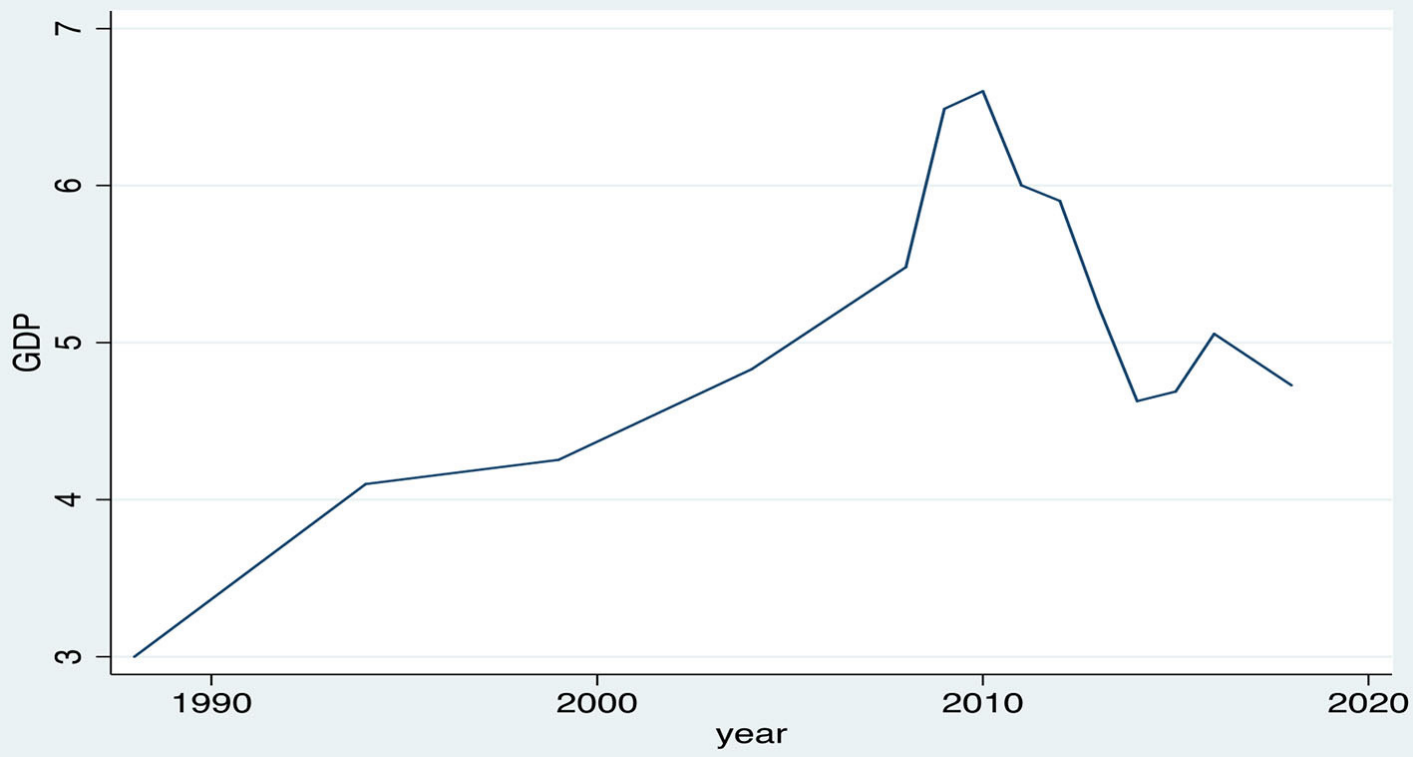
Evolution of the public health expenditure as a percentage of GDP, 1988–2018, Greece. Source: HEL.STAT. (HBS metadata, 1988-2018).

At this point, we present the findings regarding the public health expenditure as a percentage of GDP in Greece. The results of this study are illustrated in Figure 6, proving that public spending had an increasing trend within the period 1988 to 2010, when the percentage of the health expenditure to GDP rose from 3 to 6.6%, whereas from 2010 up to 2018, we observed a sharp reduction in the public spending, due to the implementation of fiscal adjustment strict measures, from 6.6 to 4.7% of GDP.

### Joint Presentation of the HHE and the Public Health Expenditure Development (Multiple Cointegration)

**Figure 7:**
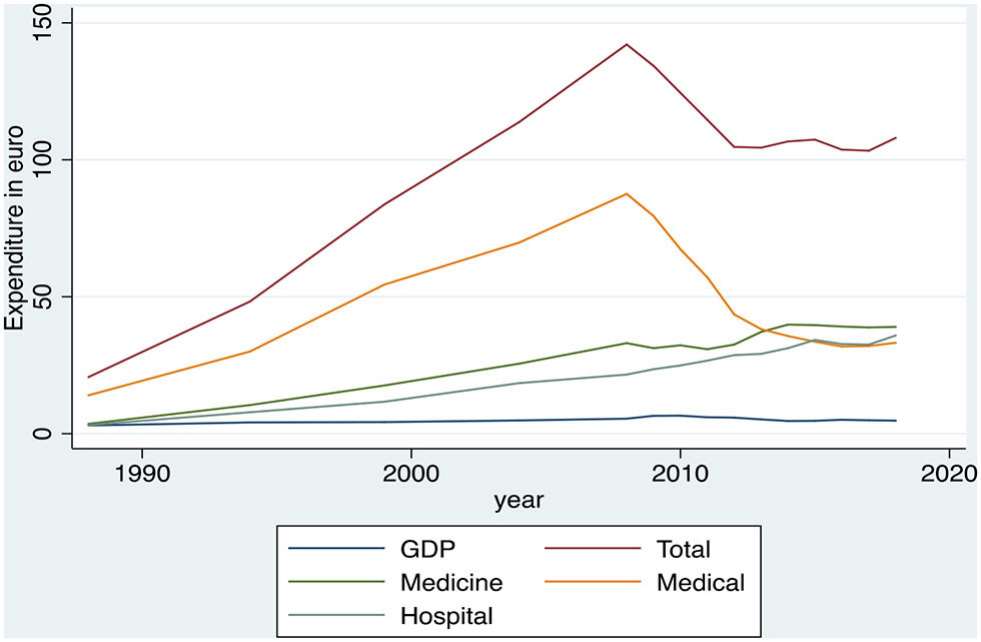
Evolution of the public health expenditure as a percentage of GDP and the Greek household health expenditure, 1988–2018. Source: OECD Health Statistics, 2019 & HEL.STAT. (HBS metadata, 1988–2018).

Figure 7 shows both, simultaneously, the HHE and the public health expenditure development. According to this graph, the private household expenditure seems to go “hand in hand” with the public expenditure, because, as mentioned previously, the total household spending appears to have a strong growth during the period 1988–2008 and a significant reduction during the period 2008–2018. The total public expenditure also shows a strong growth during the period 1988–2010 and a significant reduction in the period 2010–2018.

### Cointegration Analysis Results Between the Public Health Spending and the Greek HHE

In the last section of results, data are presented regarding the integration of the HHE to the public expenditure as a percentage of GDP. Because of few observations (which did not allow us to draw reliable conclusions), Johansen test was not expected to yield results in the total data set, and therefore, integration test between the public health expenditure and the total private health expenditure was applied (Table 5), as well as between the public health expenditure and the two main categories of the private health expenses (medical and pharmaceutical).

**Table 5:** Cointegration analysis results between the public health spending and the Greek household health expenditure.

	**Johansen tests for cointegration**					
**Trend: trend Sample: 2010–2018**						**Number of obs = 9 Lags = 2**
**Maximum rank**	**Parms**	**LL**	**Eigenvalue**	**Trace statistic**	**5% critical value**	
0	8	−19.389823		26.6515	18.17	
1	11	−7.1448956	0.93420	2.1616*	3.74	
2	12	−6.0640754	0.21352			

*Source: OECD Health Statistics, 2019 and HEL.STAT. (HBS metadata, 1988–2018)*.

Table 5 shows that for *r* = 0, the null hypothesis is rejected (because trace statistic > critical value), and this means that we reject the hypothesis of cointegration equation absence. Conversely, for *r* = 1, the null hypothesis is not rejected (2.16 < critical value = 3.74), and we do not reject the null hypothesis that there is only one cointegration equation.

This means that there is a cointegration relationship between the public health spending and the private health expenditure.

Subsequently, cointegration equation was evaluated and the multivariate model/error correction model VEC model (or otherwise ECVAR). The results are detailed in Table 6.

**Table 6:** Cointegration equation and VEC model results between the Greek public expenditure and the household health expenditure.

	**Johansen normalization restriction imposed**						
	**beta**	**Coef**.	**Std. Err**.	**z**	**P>|z|**	**(95% conf. Interval)**	
**_ce1**	V1 GDP _cons	1−22.831864.635125	1.574286	−14.50	0.000	−25.9174	−19.74632
**D_V1**							
	_ce1 L1.	−7.181892	0.290038	−2.48	0.013	−1.286653	−0.1497253
	V1 LD.	2.218084	0.6594626	3.36	0.001	0.925561	3.510607
	GDP LD.	1.826119	2.789096	0.65	0.513	−3.640409	7.292648
	_cons	−0.0001754	1.575419	−0.00	1.000	−3.08794	3.087589
**D_GDP**							
	_ce1 L1.	0.0680826	0.0089817	7.58	0.000	0.0504788	0.0856865
	V1 LD.	−0.1026407	0.0204218	−5.03	0.000	−0.1426667	−0.0626147
	GDP LD.	−0.0681388	0.086371	−0.79	0.430	−0.2374228	0.1011451
	_cons	−0.0018506	0.0487866	−0.04	0.970	−0.0974705	0.0937694

*Source: HEL.STAT (HBS metadata, 1988–2018)*.

The cointegration equation is as follows:

$$\begin{array}{l} {\text{ECT}}_{t-1}=4.635+{\text{Household HE}}_{t-1}-22.832\cdot {\text{Public HE}}_{t-1} \end{array}$$

VEC indicates that long-run equilibrium equations are as follows:

$$\begin{array}{l} \Delta {\left(\text{Public HE}\right)}_{t}=-0.718\cdot ECT_{t-1}\;+1.826\cdot {\text{Public HE}}_{t-1}\\ \;+2.218\cdot {\text{Household HE}}_{t-1}-0.0002\\ \Delta {\left(\text{Household HE}\right)}_{t}=0.681\cdot ECT_{t-1}-0.681\cdot {\text{Public HE}}_{t-1}\\ \;-0.104\cdot {\text{Household HE}}_{t-1}-0.002 \end{array}$$

The results suggest that there is a statistically significant cointegration between the public expenditure and the private household spending. The public expenditure (GDP) coefficient in the cointegration equation is statistically significant, so are the adjustment parameters. The adjustment parameters in this bilateral example are easy to be interpreted and one can notice that the estimates have the correct signs (negative) and indicate rapid adjustment toward equilibrium, showing that as the public health expenditures increase, the private expenditures are reduced (negative: *b* = −22.832, *p* < 0.05). The estimation of the coefficient D_V1 (ce1) equals −0.718 (*p* < 0.05). Therefore, when the average rate of private expenditure is very high, a sharp fall is followed to the level of the public expenditure. The estimated coefficient D_GDP (ce1) equals 0.068 (*p* < 0.05), indicating that the average rate of the public spending is quickly adapted to the levels of the private spending. This means that ~71.8% of the imbalances between the private and the public expenditure are corrected by changing the private spending, whereas only 6.8% of the imbalances are corrected by changing the public spending.

The results clearly indicate a bi-long relationship between the private and the public expenditure, where the private spending seems to be adapted more rapidly to the public spending, while there are those who mainly correct the imbalance between the private and the public expenditure.

## Discussion

It should be noted here that health expenditures in Greece are financed through a combination of funding agencies, including the public spending and the mandatory health insurance (“government/mandatory”) and the private health insurance, and the private funds, such as the out-of-pocket household payments, non-governmental organizations expenditures, and so on.

However, the Greek health care system faces a public funding gap, resulting from the current financial crisis and the relevant austerity measures being forced. Official data have shown that Greece's GDP has been contracted by 25% within the last decade, and this has had an impact on the health expenditure of the Greek households (of already high out-of-pocket payment), as well. Based on the literature review, it is clear enough that most OECD countries followed, more or less, the same route: increase in spending during pre-crisis period and declining afterward. The austerity program, in which Greece entered due to the financial crisis, has indeed significantly affected the HHE, as well. Both microeconomics and macroeconomics research lead to the same conclusion.

As mentioned before, in the research approach of the whole project, part of which is hereof, both microeconomics and macroeconomics were used. Studying the microeconomic data in the health care market of a country may provide very important information in detail. One can easily find where individuals purchase medical products or services; the decisions they make, which are based on price; quantity and quality; and how these three factors interact with each other. Then, health policy makers can perform or adjust more effectively their health care planning into the community.

Other people gloat over the fact that macroeconomic approach is always the best to adopt, as it refers to the “sum total of economic activity, dealing with the issues of growth, inflation and unemployment” (17). Macroeconomics helps countries to understand their development and growth, and in terms of health care, it influences policy making and planning in macro perspective.

The only thing beyond doubt is that the combination of macroeconomic and microeconomic studies is a complementary perspective on the overall subject of the health care economy and therefore should both be considered on decision making by health care policy planners.

## Limitations

Perhaps, the most important limitation of any study, concerning the private health expenditure, is the lack of data on the informal payments (under-the-table payments to medical staff, etc., as mentioned previously), which constitute the phenomenon of the shadow economy and make the already enormous private health costs skyrocket. Although the present study is based on the official published metadata (HBS) and not on the original data collected by the authors, while part of these informal payments is included in HBS, thus making HBS more reliable than in other surveys, in no way were they considered to be representative of the actual size of informal payments. Furthermore, presented data in this study may not fully reflect the Greek health expenditure, given the relatively short period of studying. Further research and comparative analysis are needed, to identify the impact of Greece's financial crisis on the HHE, whereas the effect of the economic crisis appears clearer over the longer periods of time. However, the statistical approach and the validation of hypothesis testing remain the strongest point of this study.

## Conclusions

The austerity program, in which Greece entered because of the financial crisis, significantly affected the health expenditure. The results of this study showed that the average total household expenditure increased 5-fold from 1988 to 2008, and from 2008 to 2018, the average total expenditure of households on health decreased by 24%.

Regarding subsets, it was shown that the average household expenditure on medicines, therapeutic appliances, and equipment were considerably increased from 1988 to 2018, while it seemed that it was the only category that was not affected significantly by the financial crisis. Similarly, the average household spending on the hospital care increased notably from 1988 to 2018, and it was affected very slightly by the economic crisis. Conversely, the average costs for medical services showed an upward trend from 1988 to 2008 and then a significant decrease from 2008 to 2018.

As regards the public expenditure, the results showed that there was an increase during the period 1988–2010 and a decrease during the period 2010–2018. In fact, one can say that the households at the onset of the financial crisis have had reduced the health expenditure, while the state has adapted to this situation 2 years later.

Lastly, the cointegration analysis showed significant cointegration between the public health expenditure and the total household expenditure and significant cointegration between the public health expenditure and the medical and pharmaceutical household expenditure.

In a review of the literature, the expansion of reliance of the health care funding on out-of-pocket payment has increased the financial risk and the hardship of the Greek households, which may disrupt their living conditions and create barriers to health care access. Cost-sharing policies should recognize the different social protection needs of the households (18). Another study also reported that the Greek HHE became more sensitive to the income changes after the introduction of the economic adjustment period (19).

Taking into account the fragile household health care behavior described herewith, on the basis that the health expenditure will sparingly begin to rise in the foreseeable future due to the severe impact of population aging, with simultaneous smaller share of active population, which means significant revenue shortfalls, it is recommended that the health policy planning should consider and obviate all these challenges by financing and designing social safety nets.

## Data Availability Statement

Publicly available datasets were analyzed in this study. This data can be found here: Greek Statistical Authority (HEL.STAT.), Micro-data in primary form on Household Budget Surveys (H.B.S.) of the years 1987/88, 1993/94, 1998/99, 2004, 2008, 2009, 2010, 2011, 2012, 2013, 2014, 2015, 2016, 2017 and 2018, on-line available at: http://www.statistics.gr/el/statistics/-/publication/SFA05/2008 (in site only from 2008 onwards. For the older H.B.S., after special permission to HEL.STAT. archives for research purposes).

## Author Contributions

All authors listed have made a substantial, direct and intellectual contribution to the work, and approved it for publication.

## Conflict of Interest

The authors declare that the research was conducted in the absence of any commercial or financial relationships that could be construed as a potential conflict of interest.

## Footnotes

^1^Every 4 years previously, on an annual basis from 2008 onward.
